# Associations between severe and notifiable respiratory infections during the first trimester of pregnancy and congenital anomalies at birth: a register-based cohort study

**DOI:** 10.1186/s12884-023-05514-8

**Published:** 2023-03-24

**Authors:** Abrar A. Chughtai, Wen-Qiang He, Bette Liu

**Affiliations:** School of Population Health, University of New South Wales, Samuels Building, Kensington Campus, Sydney, NSW 2052 Australia

**Keywords:** Respiratory infections, Influenza, Congenital anomalies, Birth defects, Cardiac anomalies

## Abstract

**Background:**

Evidence regarding the association between acute respiratory infections during pregnancy and congenital anomalies in babies, is limited and conflicting. The aim of this study was to examine the association between acute respiratory infections during the first trimester of pregnancy and congenital anomalies in babies using record linkage.

**Methods:**

We linked a perinatal register to hospitalisation and disease notifications in the Australian state of New South Wales (NSW) between 2001 to 2016. We quantified the risk of congenital anomalies, identified from the babies’ linked hospital record in relation to notifiable respiratory and other infections during pregnancy using generalized Estimating Equations (GEE) adjusted for maternal sociodemographic and other characteristics.

**Results:**

Of 1,453,037 birth records identified from the perinatal register between 2001 and 2016, 11,710 (0.81%) mothers were hospitalised for acute respiratory infection, 2850 (0.20%) had influenza and 1011 (0.07%) had high risk infections (a record of cytomegalovirus, rubella, herpes simplex, herpes zoster, toxoplasmosis, syphilis, chickenpox (varicella) and zika) during the pregnancy. During the first trimester, acute respiratory infection, influenza and high-risk infections were reported by 1547 (0.11%), 399 (0.03%) and 129 (0.01%) mothers. There were 15,644 (1.08%) babies reported with major congenital anomalies, 2242 (0.15%) with cleft lip/ plate, 7770 (0.53%) with all major cardiovascular anomalies and 1746 (0.12%) with selected major cardiovascular anomalies. The rate of selected major cardiovascular anomalies was significantly higher if the mother had an acute respiratory infection during the first trimester of pregnancy (AOR 3.64, 95% CI 1.73 to 7.66). The rates of all major congenital anomalies and all major cardiovascular anomalies were also higher if the mother had an acute respiratory infection during the first trimester of pregnancy, however the difference was no statistically significant. Influenza during the first trimester was not associated with major congenital anomalies, selected major cardiovascular anomalies or all major cardiovascular anomalies in this study.

**Conclusion:**

This large population-based study found severe acute respiratory infection in first trimester of pregnancy was associated with a higher risk of selected major cardiovascular anomalies in babies. These findings support measures to prevent acute respiratory infections in pregnant women including through vaccination.

**Supplementary Information:**

The online version contains supplementary material available at 10.1186/s12884-023-05514-8.

## Background

The World Health Organization estimates that each year around 7.9 million infants (6% of total global births) are born with one or more congenital anomalies and of those around 300,000 die within 4 weeks of birth [1]. The estimated prevalence of major congenital anomalies is 2.8 to 3% of live births, but reported rates vary due to different classifications of major congenital anomalies [2]. The most commonly reported major congenital anomalies are cardiac, neural tube and Down syndrome. The aetiology of many congenital anomalies remains unclear. The main causes are believed to be genetic (10–30%), environmental (5–10%), inherited (20–35%), with about 30–45% remaining unidentified [3]. Maternal risk factors include, age, genetic predisposition, exposure to teratogens during pregnancy (e.g. alcohol, smoking, drugs, chemicals), infection and other maternal conditions, such as diabetes and thyroid disorders [4–6].

Some infections, particularly during the first trimester of pregnancy, are known to cause congenital anomalies in babies [7]. These are frequently referred to as TORCH (Toxoplasmosis, Other (syphilis, varicella-zoster, parvovirus B19), Rubella, Cytomegalovirus (CMV), and Herpes virus) [8, 9] but other infections such as the Zika virus are known to cause congenital anomalies [10]. Infections during pregnancy also have the potential for transmission to the foetus or to the new born babies at the time of or immediately after delivery, and hence can have serious implications [11].

Acute respiratory infections are commonly reported in pregnant women [12–14] and there is some evidence that acute respiratory infections, particularly influenza, in pregnant women may be associated with congenital anomalies in babies, however the evidence is limited and conflicting [13, 15–18]. Some studies showed an association of maternal respiratory infections during the first trimester with all congenital anomalies in babies [15, 19], while others showed an association with selected congenital anomalies such as oesophageal atresia/stenosis [13], left obstructive cardiac defects [17], spina bifida, hydrocephalus, anorectal malformations [18] and cleft lip/ palate [20]. Influenza infection in the first trimester of pregnancy has also been found to be associated with increased risk of non-chromosomal congenital anomalies, like neural tube anomalies, hydrocephaly, congenital heart anomalies, cleft lip, digestive system anomalies and limb reduction anomalies [16, 21–23]. Febrile illness and hyperthermia in early pregnancy was also associated with an increased risk of cardiac [17] and neural tube defects, including when it is due to influenza [24, 25]. However, most of these studies examined association of respiratory infections (or influenza) with specific congenital anomalies as the underlying biological mechanisms of various congenital anomalies are different [26].

As most studies have been case control or cross-sectional design with many also using self-reported infection, they may be subject to recall bias [13, 17–19, 21, 27]. Therefore, the aim of our study was to examine associations between respiratory infections during the first trimester of pregnancy and subsequent major congenital anomalies in babies in New South Wales (NSW), using a large perinatal dataset and record linkage.

## Methods

### Study population

This study was conducted by probabilistically linking records from three statutory databases in the Australian state of New South Wales (NSW). The NSW Perinatal data collection (PDC) was linked to the Admitted Patients Data Collection (APDC) and the Notifiable Conditions Information System (NCIMS) [28]. The data were linked by the NSW Centre for Health Record Linkage (CHeReL) with false-positive and false-negative linkage rates reported to be < 0.5% [29].

The PDC is a population-based surveillance system covering all live births and stillbirths of at least 20 weeks gestation or at least 400 g birthweight in NSW public and private hospitals, as well as homebirths. It collects data on patterns of pregnancy care and pregnancy outcomes and contains records of both the mothers and the babies. Terminations of pregnancy for fetal anomalies before 20 weeks of gestation are not included in PDC. The APDC records all inpatient separations (discharges, transfers, and deaths) from all public, private, psychiatric and repatriation hospitals in NSW. Records include a date of admission and information on the reasons for admission coded according to the International Classification of Diseases 10th Revision – Australian Modification (ICD-10-AM). The NSW NCIMS is a register of diagnoses of certain infectious diseases and adverse events following immunisation, notified to the NSW Department of Health by laboratories, hospitals, medical practitioners, schools, and child care centres [28]. The study population included all mothers having a singleton birth and their babies in the PDC between 2001 to 2016.

### Outcome and exposure variables

The primary outcome measure was a major congenital anomaly defined if a baby with a birth record in the PDC linked to an APDC record coded for a congenital anomaly. For this study, we used ICD-10-AM codes that were consistent with national Australian classification of major congenital anomalies [30]. We also examined cleft lip/palate and selected major cardiovascular anomalies based on Australian reporting (hereafter “selected major cardiovascular anomalies”) [30] and all major cardiovascular anomalies separately (ICD -AM codes Q20 to Q25). See supplementary Table S1 for codes.

The main exposures of interest were influenza and other acute respiratory infections in the mother during the first trimester of pregnancy. The gestational age (length of pregnancy) was calculated from the PDC Register, based on the clinical estimate either from an ultrasound or last menstrual period. The first trimester exposure included exposure in mother from start of gestational week 1 to the end of week 12. As a comparator, we also analysed acute respiratory infections in the mother during the 2nd / 3rd trimester of pregnancy and high-risk infections in pregnancy, known to cause congenital anomalies (hereafter “high-risk infections”). These conditions included cytomegalovirus, rubella, herpes simplex, herpes zoster, toxoplasmosis, syphilis, chickenpox (varicella), zika. These infection groups were identified from linked APDC (based on ICD-10-AM primary or secondary diagnosis codes) or NCIMS records. We included both laboratory and clinically confirmed infections in this study. All respiratory infections (ICD-10 AM code J00-J06, J09-J18, J20-J22), including bacterial respiratory infections were included. Classification is done in collaboration between clinicians and clinical coders. At the time of discharge, a clinical coder reviews the patient’s healthcare record and abstracts information recorded by clinicians to assign codes for the principal diagnosis, additional diagnoses and interventions performed [31, 32]. Details of data sources and codes used for each infection group are listed in supplementary Table S2. We classified an infant as exposed to one of these infection groups if their mothers record linked to an APDC or NCIMS record dated as occurring during their pregnancy.

### Analysis

As a woman can have multiple records of births in the PDC, generalized Estimating Equations (GEE) of logit link function with an exchangeable correlation structure were used to estimate associations of each exposure of interest: acute respiratory tract infection, influenza and high-risk infections in pregnancy, with the subsequent likelihood of congenital anomalies (including major anomalies, major cardiovascular anomalies and cleft lip/ palate) in their infants. Crude and adjusted odds ratios (ORs) were calculated from the GEE models with corresponding 95% confidence intervals. All known and potential confounders identified through the literature were included in the final model, including maternal age group at delivery (< 20, 20–24, 25–29, 30–35, 36–39 and ≥ 40 years), smoking during pregnancy (yes, no), remoteness of area of residence (major city, inner regional, outer regional/ remote), quartile of socioeconomic status based on area of residence (Q1 most disadvantaged, Q2, Q3, Q4 least disadvantaged), previous pregnancy (yes, no), country of birth (Australia, overseas), hospital of delivery (public, private hospital unknown), the number of weeks pregnant at first antenatal visit (0–13, 14–25, 26–45), Indigenous status (non-Indigenous, Indigenous), hypertension (yes, no), and pregestational /gestational diabetes (yes, no). These data were collected as part of routine reporting to the perinatal data collection and were included a priori. All analyses were conducted using SAS proc. GENMOD. All statistical tests were two sided, and a *P* < .05 was considered statistically significant.

### Ethics approval

Ethical approval was obtained from the New South Wales Population Health Research Ethics committee: HREC/09/CIPHS/71.

## Results

A total of 1,453,037 birth records were identified from the PDC during the study period (2001 to 2016). At the time of giving birth most mothers were aged between 20 to 39 years (92.5%), were non-smokers (87.6%) and were living in major cities (79.7%). More than two thirds of mothers (68.2%) were born in Australia and less than a quarter of the total births (22.3%) occurred in private hospitals. A total of 12,580 (0.87%) mothers had hypertension and 97,677 (6.7%) had diabetes during the pregnancy (Table 1). Among mothers, 11,710 (0.81%) had linked to a record documenting an acute respiratory infection, 2850 (0.20%) had influenza and 1011 (0.07%) had ‘high risk infections’ during the pregnancy. Of all those with an acute respiratory infection, influenza and high-risk infections, 72.5% (8489/11,710), 20% (570/2850) and 85% (859/1011) respectively were solely identified from hospitalisation records. During the first trimester of pregnancy, acute respiratory infection, influenza and high-risk infections were reported by 1547 (0.11%), 399 (0.03%) and 129 (0.01%) mothers. Characteristics of women with and without acute respiratory infections and influenza are presented in supplementary Tables S3a and b.

**Table 1:** Characteristics of women giving birth in New South Wales Australia from 2001 to 2016

Variables	Proportion	Number
**Age**		
< 20	3.4	48,801
20–24	13.4	194,923
25–29	27.3	396,413
30–35	33.5	486,675
35–39	18.3	266,404
40+	4.1	59,821
**Smoking status**		
No	87.6	1,272,641
Yes	12.2	177,994
Unknown	0.2	2402
**Remoteness of area of residence**		
Major city	79.7	1,157,831
Inner regional	14.7	214,217
Outer regional/Remote	4.4	63,301
Unknown	1.2	17,688
**Socioeconomic status of area of residence**		
Q1 Most disadvantaged	24.2	352,279
Q2	24.2	352,229
Q3	24.3	352,665
Q4 Least disadvantaged	26.0	378,174
Unknown	1.2	17,690
**Previous pregnancy**		
No	42.6	619,637
Yes	57.3	832,560
Unknown	0.1	840
**Hospital of delivery**		
Public	77.7	1,129,486
Private	22.3	323,496
**Maternal country of birth**		
Australia	68.2	991,152
Overseas	31.8	461,885
**Weeks at first antenatal visit**		
0–13	68.4	993,634
14–25	25.8	375,509
26–45	4.6	66,831
Unknown	1.2	17,063
**Indigenous status**		
Non-indigenous	96.6	1,404,035
Indigenous	3.2	46,531
Unknown	0.2	2471
**Hypertension**		
No	99.1	1,440,457
Yes	0.9	12,580
**Diabetes**		
No	93.3	1,355,360
Yes	6.7	97,677

Of the 1,453,037 births, 15,644 (1.08%) babies were born with major congenital anomalies, 2242 (0.15%) were born with cleft lip/ plate, 1746 (0.12%) were born with selected major cardiovascular anomalies, and 7770 (0.53%) were born with any major cardiovascular anomaly. Of those with a major congenital anomaly, 1.45% (229) had a concurrent genetic condition.

During the first trimester, only selected major cardiovascular congenital anomalies was significantly associated with acute respiratory infections in multivariate analysis. The odds of developing selected major cardiovascular congenital anomalies were 3 time higher if the mother had an acute respiratory infection during the first trimester of pregnancy (AOR 3.64, 95% CI 1.73 to 7.66). Rate of selected major cardiovascular anomalies were also higher in babies if mother had influenza during the first trimester of pregnancy, however study was under powered to detect statistically significant difference (OR 2.04, 95% CI 0.29 to 14.42) (Tables 2 and S4 and S5).

**Table 2:** Rate of congenital anomalies according to type of infection during the first trimester of pregnancy and odds ratios for association^a^

	n/N (%)	OR (95% CI)	Adjusted OR (95% CI)^b^
**Major congenital anomalies**			
**Acute respiratory infection**			
No	15,486/1441327 (1.07)	Ref	Ref
Yes	22/1547 (1.42)	1.33 (0.88, 2.03)	1.33 (0.88, 2.02)
**Influenza**			
No	15,612/1450187 (1.08)	Ref	Ref
Yes	4/399 (1.00)	0.94 (0.35, 2.5)	0.95 (0.36, 2.54)
**High-risk infections**^c^			
No	15,630/1452026 (1.08)	Ref	Ref
Yes	1/129 (0.78)	0.71 (0.10, 5.20)	0.72 (0.1, 5.27)
**Cleft lip and palate**			
**Acute respiratory infection**			
No	2222/1441327 (0.15)	Ref	Ref
Yes	1/1547 (0.06)	0.43 (0.06, 2.91)	0.43 (0.06, 2.9)
**Influenza**			
No	2239/1450187 (0.15)	Ref	Ref
Yes	1/399 (0.25)	1.62 (0.22, 11.66)	1.68 (0.23, 12.18)
**High-risk infections**^b^**/**			
No	2240/1452026 (0.15)		
Yes	0/129 (0)		
**Selected cardiovascular anomalies**^d^			
**Acute respiratory infection**			
No	1724/1441327 (0.12)	Ref	Ref
Yes	7/1547 (0.45)	**3.79 (1.80, 7.99)**	**3.64 (1.73, 7.66)**
**Influenza**			
No	1739/1450187 (0.12)	Ref	Ref
Yes	1/399 (0.25)	2.10 (0.3, 14.86)	2.04 (0.29, 14.42)
**High-risk infections**^c^			
No	1745/1452026 (0.12)		
Yes	0/129 (0)		
**All major cardiovascular anomalies (ICD AM Q20–25)**			
**Acute respiratory infection**			
No	7671/1441327 (0.53)	Ref	Ref
Yes	14/1547 (0.90)	1.70 (1.00, 2.89)	1.68 (0.99, 2.84)
**Influenza**			
No	7745/1450187 (0.53)	Ref	Ref
Yes	2/399 (0.50)	0.95 (0.24, 3.75)	0.95 (0.24, 3.78)
**High-risk infections**^c^			
No	7758/1452026 (0.53)	Ref	Ref
Yes	1/129 (0.78)	1.45 (0.20, 10.45)	1.41 (0.19, 10.26)

BOLD type indicates *p* < 0.05

^a^Only first trimester analysis is included in the Table 2. We have kept infections in 2nd/3rd trimester in analyses as a separate group, but they are not shown in the table. Results of 2nd/3rd trimesters are presented in the supplementary tables (S4, S5 and S6)

^b^Adjusted for maternal age group at delivery, smoking during pregnancy, remoteness of area of residence, quartile of socioeconomic status based on area of residence, previous pregnancy, country of birth, hospital of delivery, the number of weeks pregnant at first antenatal visit, Indigenous status, hypertension, and diabetes

^c^TORCH and other known causes of congenital anomalies

^d^As per Australian reporting - Abeywardana S & Sullivan EA 2008. Congenital anomalies in Australia 2002–2003. Birth anomalies series no. 3 Cat. no. PER 41. Sydney: AIHW National Perinatal

The rate of all major congenital anomalies in babies was 1.42% (22/1547) if the mother had an acute respiratory infection during the pregnancy and 1.07% (15,486/1441327) if mother did not have an acute respiratory infection during first trimester of pregnancy, however difference was not statistically significant (AOR 1.33, 95% CI 0.88 to 2.02). The rate of major congenital anomaly was 1% in infants of women with an influenza diagnosis during the first trimester compared to 1.08% in those without a diagnosis, but again this was not statistically different in the adjusted analysis (AOR 0.95, 95% CI 0.36 to 2.54). The corresponding rates for women with high-risk infections during the first trimester of pregnancy compared to those without was: 0.78% vs 1.08%, (AOR 0.72, 95% CI 0.01 to 5.27) (Table 2, and S4, S5 and S6).

Rates of all major cardiac anomalies in babies born to women who had acute respiratory infections, influenza or high-risk infections during the first trimester of pregnancy, were 0.90%, 0.50 and 0.78% respectively. After adjustments these rates were not significantly different in babies born to women without acute respiratory infections (AOR 1.68, 95% CIs 0.99 to 2.84), influenza (AOR 0.95, 95% CI 0.24 to 3.78) and high-risk infections (AOR 1.41, 95% CI 0.19 to 10.26) during pregnancy. Likewise, cleft lip/ cleft palate was also not associated with acute respiratory infections, influenza or high-risk infections in mother during the first trimester of pregnancy (Table 2, and S4, S5 and S6.

## Discussion

To our knowledge, this is the first study to examine associations between respiratory infections in pregnant women during the first trimester and major congenital anomalies in their babies using large population based linked datasets. We reported significantly higher rates of selected major cardiovascular anomalies in babies born to women who had acute respiratory infections during pregnancy. Rates of major congenital anomalies and all major cardiovascular anomalies were also higher in babies born to women who had acute respiratory infections during the first trimester of pregnancy, however study was underpowered to detected statistically significant difference. Large scale studies should be conducted to confirm association between laboratory confirmed respiratory infection with major congenital anomalies and major cardiovascular anomalies. Pregnant women should be aware of this risk and to use vaccines and other preventive measures to protect from respiratory infections during pregnancy.

Previous studies have also found an association of acute respiratory infections during early pregnancy with major cardiovascular anomalies [23]. Xia et al. examined the associations between maternal upper respiratory tract infection during the first trimester of pregnancy and the risk of congenital cardiac anomalies in babies through a hospital-based case-control study and a meta-analysis involving 11,911 cases and 74,358 controls from 16 case control studies [23]. They found an association of upper respiratory tract infection during pregnancy with all congenital heart diseases [23]. Both simple and complex congenital cardiac anomalies were included in the study. Like previous studies [15], we also reported higher rates of major congenital anomalies in babies born to women who had acute respiratory infections during the first trimester of pregnancy, however study was underpowered to detected statistically significant difference, which is likely due to not including terminations of pregnancy for fetal anomalies before 20 weeks of gestation. A large population-based cross-sectional survey in pregnant Chinese women found an increased risk of major congenital anomalies in babies following respiratory infections in mothers during the pregnancy [15]. However, unlike our study, this cross-sectional study may be prone to differential reporting of respiratory infections. Maternal common cold was also reported to be associated with congenital anomalies in a study, though both major and minor congenital anomalies were included [20].

Most of the previous studies report a relationship of respiratory infections during the first trimester of pregnancy with selected congenital anomalies [13, 15–17, 23]. Acs et al. conducted a case control study and included 22,843 cases with congenital abnormalities, 38,151 population controls without congenital abnormalities and 834 controls with Down syndrome. Compared with population controls, acute respiratory infections during the first trimester of pregnancy in case mothers was associated with esophageal atresia/stenosis, posterior cleft palate, exomphalos/gastroschisis, diaphragmatic anomalies, multiple anomalies, cleft lip ± palate and poly/syndactyly. While comparing cases to controls with Down syndrome, only esophageal atresia/stenosis was significantly associated with acute respiratory infections during pregnancy [13]. Zhang et al. conducted a case control study using Shanghai Birth Defects Monitoring Program and reported an association between common cold in first trimester of pregnancy and major congenital anomalies of nervous system in babies including anencephalus, spina bifida and hydrocephalus [20]. Another case control study in China found an association between maternal upper respiratory tract infection during the first trimester of pregnancy with anorectal malformation in babies (AOR 2.44, 95% CI 1.29–4.63) [18]. Although we also reported high increased risk of selected major cardiovascular anomalies after acute respiratory infections in the first trimester, we did not examine the risk associated with isolated anomalies due to small sample size. Unlike these studies, we grouped congenital anomalies into major anomalies and major cardiovascular anomalies to increase statistical power and only examined the cleft lip/ palate as isolated anomaly, which was not significant.

Previous studies have demonstrated an association between influenza and congenital cardiac anomalies [16, 21–23]. Luteijn et al. conducted a meta-analysis of 13 case control studies and 9 cohort studies [16]. The study found an association of influenza in pregnancy with any congenital anomaly with the odds of developing any congenital anomaly two times higher if the mother had influenza during the first trimester of pregnancy [16]. While we observed higher rates of selected major cardiovascular anomalies following influenza in pregnant women, the difference was statically significant in adjusted analyses. Acs et al. reported an association of maternal influenza during the first trimester of pregnancy with cardiovascular malformations [21]. Like our study, Acs et al. also included medically recorded influenza cases in the analysis. In another study, influenza during pregnancy was not associated with all cardiac anomalies, but some selected cardiac defects such as left obstructive defects (Hypoplastic left heart, Aortic stenosis and Aortic coarctation) right obstructive defects (Tricuspid atresia) and ventricular septal defect [17]. However, this study used self-reported influenza like illness while we only included medically reported cases. A US population-based case-control study also reported an association of influenza in women during pregnancy and right-sided obstructive defects (Tricuspid atresia and Pulmonary atresia), but not with congenital heart diseases in aggregate [27].

Congenital anomalies after influenza and other respiratory infections may be due to multiple direct or indirect pathways, including transplacental infection of the foetus or the indirect effect of fever induced toxic metabolites or drugs used to treat these infections [33, 34]. Hyperthermia impairs protein synthesis and causes cell death, resulting in congenital anomalies [35]. Moreover, in hyperthermia, there are metabolic changes in the body, and these metabolites may cross the placenta to cause direct damage to the embryo [34]. A meta-analysis showed that there was two times more risk of neural tube anomalies in infants whose mothers had hyperthermia [25]. A relation between maternal hyperthermia and congenital cardiac anomalies and orofacial clefts has also been established [27, 36–38]. Additionally, maternal fever from influenza during early pregnancy was found to be related to heightened risk for selected congenital anomalies- anencephaly, spina bifida, encephalocele, cleft lip with or without cleft palate, colonic atresia/stenosis, bilateral renal agenesis/hypoplasia, limb reduction anomalies, and gastroschisis [39]. Moreover some drugs used to treat hyperthermia and infections during the first trimester also cause congenital anomalies in babies [40, 41]. Like previous studies, we reported increased risk of congenital anomalies after respiratory infections with the risk appearing to be greatest in the first trimester [16, 20], however we did not have information on fever or drugs used during pregnancy in this study.

The strengths of the study are the large population-based linked dataset and the good validity of data on infection and congenital anomalies as this information was taken from hospital records or disease notifications. We only used hospital records for ascertainment of congenital anomalies, so only confirmed cases are included. The sensitivity of APDC diagnostic codes to detect major congenital anomalies has been reported from 80 to 95%, however it varies by condition reported [42], but notably the sensitivity of the APDC to detect major congenital anomalies included in this analysis has been shown to be high (85.0, 95% CI 83.7–86.3)” [43]. Although we included both laboratory and clinically confirmed infections in this study, our ascertainment of infections was based on laboratory notifications and coded hospital discharge records. Therefore, sensitivity will be high (few false positives) as sensitivity of the hospital discharge codes in Australia is reported high [44, 45]. However, these methods may not capture mild infections where no testing nor hospitalisation has occurred. Moreover, it is also possible women who were hospitalised with infections may be more likely to have other underlying conditions that made them more likely to get infection, or have infections reported, and also more likely to have congenital anomalies.

There are limitations of this study. We only included live births and stillbirths of at least 20 weeks gestation or at least 400 g and did not include termination of pregnancies for fetal anomalies before 20 weeks and spontaneous losses before 20 weeks, which might result in underreporting of congenital anomalies. Therefore, this study would miss certain severe anomalies where there is a high rate of pregnancy termination. Previous studies on the relationship between other established risk factors and congenital anomalies reported low risk of bias if the analysis was restricted to livebirths, except for congenital anomalies with high mortality risks such as anencephaly [46, 47]. Another limitation of this study was including only major congenital and major cardiovascular anomalies in the analysis and not analysing congenital anomalies in different organs separately. Biological mechanism for congenital anomalies in different organs might be different so ideally, congenital anomalies in different organs should be analysed separately. However, the number of congenital anomalies in different organs was small in our dataset so we only analysed cleft lip and cleft palate separately. Furthermore our results may not necessarily be comparable with others as there is no standard definition of major congenital anomalies, and this differs between studies [48]. Finally, we lacked comprehensive data on potential confounders such as fever, nutritional deficiencies, and alcohol use and other medical and treatment histories, including vaccination. Also observational studies may be prone to residual confounding as those with infections differed from those without infections on some characteristics that may also be associated with congenital anomalies. This lack of comprehensive data may lead to an overestimate of risks.

## Conclusion

This study adds to the growing evidence that respiratory infections during the first trimester of pregnancy can lead to subsequent major cardiovascular anomalies in babies. While more studies are required to confirm our findings, ideally prospective studies with doctor diagnosed or laboratory confirmed infections, the findings support advice to women to avoid respiratory and other high-risk infections during pregnancy and to use preventive measures to protect from respiratory infections including vaccination against respiratory pathogens.

## Supplementary Information

**Additional file 1: Supplementary Table S1.** Types of congenital anomalies included in the study, with ICD − 10 AM codes. **Supplementary Table S2.** Study variables and data sources. **Supplementary Table S3.** a: Characteristics of women with and without acute respiratory infections giving birth in New South Wales Australia from 2001 to 2016. b: Characteristics of women with and without influenza giving birth in New South Wales Australia from 2001 to 2016. **Supplementary Table S4.** Association of acute respiratory infections during the pregnancy with various congenital anomalies. **Supplementary Table S5.** Association influenza during the pregnancy with various congenital anomalies. **Supplementary Table S6.** Association of high risk infections* during the pregnancy with various congenital anomalies.

## Data Availability

All data generated or analysed during this study are included in this published article.
