# Supplementary material for: Associations between severe and notifiable respiratory infections during the first trimester of pregnancy and congenital anomalies at birth: a register-based cohort study
Source: BMC Pregnancy Childbirth. 2023 Mar 24;23:203. doi: 10.1186/s12884-023-05514-8 (PMC10037767; doi:10.1186/s12884-023-05514-8)
Supplement: Supplementary file 1 — Additional file 1: Supplementary Table S1. Types of congenital anomalies included in the study, with ICD − 10 AM codes. Supplementary Table S2. Study variables and data sources. Supplementary Table S3. a: Characteristics of women with and without acute respiratory infections giving birth in New South Wales Australia from 2001 to 2016. b: Characteristics of women with and without influenza giving birth in New South Wales Australia from 2001 to 2016. Supplementary Table S4. Association of acute respiratory infections during the pregnancy with various congenital anomalies. Supplementary Table S5. Association influenza during the pregnancy with various congenital anomalies. Supplementary Table S6. Association of high risk infections* during the pregnancy with various congenital anomalies. [file 12884_2023_5514_MOESM1_ESM.docx]

**SUPPLEMENTARY TABLES**

**Supplementary Table S1: Types of congenital anomalies included in the study, with ICD -10 AM codes**

| **Outcome variables** | **Definition** | **ICD -10 AM code** |
| --- | --- | --- |
| Major congenital anomalies according to Australian reporting* | Anencephaly | Q00.0–Q00.2 |
|  | Encephalocele | Q01.0- Q01.2, Q01.8, Q01.9 |
|  | Spina Bifida | Q05.0–Q05.9 |
|  | Microcephaly | Q02 |
|  | Arhinencephaly/ Holoprosencephaly | Q04.1, Q04.2 |
|  | Hydrocephalus | Q03.0 & Q03.1 & Q03.8 and Q03.9 |
|  |  |  |
|  | Anophthalmos/ Microphthalmos | Q11.0 & Q11.2 |
|  | Microtia | Q17.2 |
|  | Transposition of great vessels, includes double outlet ventricle | Q20.1 & Q20.3 & Q20.5 |
|  | Tetralogy of Fallot | Q21.3 |
|  | Hypoplastic left heart syndrome | Q23.4 |
|  | Coarctation of the aorta | Q25.1 |
|  | Choanal atresia | Q30.0 |
|  | Cleft palate without cleft lip | Q35.0–Q35.9 |
|  | Cleft lip with or without cleft palate | Q36.0, Q36.1, Q36.9, Q37.0–Q37.5, Q37.8, Q37.9 |
|  | Oesophageal atresia/stenosis | Q39.0–Q39.3 |
|  | Small intestinal atresia/stenosis | Q41.0-Q41.2 |
|  | Anorectal atresia/stenosis | Q42.0–Q42.3 |
|  | Hirschsprung’s disease | Q43.1 |
|  | Hypospadias | Q540–Q543, Q548, Q549 |
|  | Epispadias | Q64.0 |
|  | Renal agenesis/dysgenesis | Q60.0-Q60.6 |
|  | Cystic kidney | Q61.0-Q61.5 |
|  | Bladder exstrophy | Q64.1 |
|  | Polydactyly | QQ69.0-Q69.2, Q69.9 |
|  | Limb reduction anomalies | Q71.0–Q71.9, Q72.0–Q72.9, Q73.0, Q73.1, Q73.8 |
|  | Diaphragmatic hernia | Q79.0 |
|  | Exomphalos | Q79.2 |
|  | Gastroschisis | Q79.3 |
| Cleft lip/ palate | Cleft palate without cleft lip  Cleft lip with or without cleft palate | Q35 to Q37 |
| Selected major cardiovascular anomalies according to Australian reporting* | Transposition of great vessels, includes double outlet ventricle | Q20.1 & Q20.3 & Q20.5 |
|  | Tetralogy of Fallot | Q21.3 |
|  | Hypoplastic left heart syndrome | Q23.4 |
|  | Coarctation of the aorta | Q25.1 |
| All major cardiovascular anomalies | Congenital malformations of cardiac chambers and connections | Q20.0 to Q20.6 & Q20.8 & Q20.9 |
|  | Congenital malformations of cardiac septa | Q21.0 to Q21.4 & Q21.8 & Q21.9 |
|  | Congenital malformations of pulmonary and tricuspid valves | Q22.0 to Q22.6 & Q22.8 & Q22.9 |
|  | Congenital malformations of aortic and mitral valves | Q23.0 to Q23.4 & Q23.8 & Q23.9 |
|  | Other congenital malformations of heart | Q24.0 to Q24.6 & Q24.8 & Q24.9 |
|  | Congenital malformations of great arteries (excluding patent ductus arteriosus) | Q25.1 to Q25.9 |

** As per Australian reporting - Abeywardana S & Sullivan EA 2008. Congenital anomalies in Australia 2002–2003. Birth anomalies series no. 3 Cat. no. PER 41. Sydney: AIHW National Perinatal Statistics Unit.*

**Supplementary Table S2: Study variables and data sources**

| **Infections** | **Condition to include** | **Variables and data source** |
| --- | --- | --- |
| Acute respiratory infection **(ARI)** | Influenza, Influenza A - H1N1, Influenza A – NOS, Influenza B, Influenza A - H3N2, Influenza A - H3, Influenza – NOS, Influenza A - H1, Influenza A&B, B. Whooping cough - B. pertussis, pertussis, Pneumococcal disease (invasive) - S. pneumoniae, measles, Pneumococcal Disease (Invasive) | NCIMS |
|  | All respiratory: J00-J06, J09-J18, J20-J22 | APDC |
| Influenza | Influenza, Influenza A - H1N1, Influenza A – NOS, Influenza B, Influenza A - H3N2, Influenza A - H3, Influenza – NOS, Influenza A - H1, Influenza A&B | NCIMS |
|  | J09-J11 | APDC |
| Known infections that cause congenital anomalies (referred in this paper as high-risk infections) | Cytomegalovirus (CMV) B25, rubella B06, herpes simplex B00, herpes zoster B02, toxoplasmosis B58, syphilis A51, A52, A53, chickenpox (varicella) B01, Zika U06 | NCIMS |
|  | Cytomegalovirus (CMV) B25, rubella B06, herpes simplex B00, herpes zoster B02, toxoplasmosis B58, syphilis A51, A52, A53, chickenpox (varicella) B01, Zika U06 | APDC |

**Supplementary Table S3a: Characteristics of women with and without acute respiratory infections giving birth in New South Wales Australia from 2001 to 2016**

|  | **Acute respiratory infections (ARI)** | | |
| --- | --- | --- | --- |
|  | **Yes**  **Number (%)** | **No**  **Number (%)** | **P value** |
| Age |  |  | <0.001 |
| 10-19 | 682 (5.8) | 48119 (3.3) |  |
| 20-24 | 1889 (16.1) | 193034 (13.4) |  |
| 25-29 | 2950 (25.2) | 393463 (27.3) |  |
| 30-35 | 3470 (29.6) | 483205 (33.5) |  |
| 35-39 | 2150 (18.4) | 264254 (18.3) |  |
| 40+ | 569 (4.9) | 59252 (4.1) |  |
| Smoking status |  |  | <0.001 |
| No | 9352 (79.9) | 1263289 (87.6) |  |
| Yes | 2337 (20) | 175657 (12.2) |  |
| Unknown | 21 (0.2) | 2381 (0.2) |  |
| Remoteness of area of residence |  |  | <0.001 |
| Major city | 8807 (75.2) | 1149024 (79.7) |  |
| Inner regional | 2099 (17.9) | 212118 (14.7) |  |
| Outer regional/Remote | 725 (6.2) | 62576 (4.3) |  |
| Unknown | 79 (0.7) | 17609 (1.2) |  |
| Socioeconomic status of area of residence |  |  | <0.001 |
| Q1 Most disadvantaged | 3201 (27.3) | 349078 (24.2) |  |
| Q2 | 3214 (27.4) | 349015 (24.2) |  |
| Q3 | 2663 (22.7) | 350002 (24.3) |  |
| Q4 Least disadvantaged | 2552 (21.8) | 375622 (26.1) |  |
| Unknown | 80 (0.7) | 17610 (1.2) |  |
| Previous pregnancy |  |  | <0.001 |
| No | 4073 (34.8) | 615564 (42.7) |  |
| Yes | 7631 (65.2) | 824929 (57.2) |  |
| Unknown | 6 (0.1) | 834 (0.1) |  |
| Hospital of delivery |  |  | <0.001 |
| Public | 9853 (84.1) | 1119633 (77.7) |  |
| Private | 1855 (15.8) | 321641 (22.3) |  |
| Unknown | 2 (0) | 53 (0) |  |
| Maternal country of birth |  |  | <0.001 |
| Australia | 8886 (75.9) | 982266 (68.2) |  |
| Overseas | 2824 (24.1) | 459061 (31.8) |  |
| Weeks at first antenatal visit |  |  | 0.743 |
| 0-13 | 8041 (68.7) | 985593 (68.4) |  |
| 14-25 | 3013 (25.7) | 372496 (25.8) |  |
| 26-45 | 524 (4.5) | 66307 (4.6) |  |
| Unknown | 132 (1.1) | 16931 (1.2) |  |
| Indigenous status |  |  | <0.001 |
| Non-indigenous | 10893 (93) | 1393142 (96.7) |  |
| Indigenous | 801 (6.8) | 45730 (3.2) |  |
| Unknown | 16 (0.1) | 2455 (0.2) |  |
| Hypertension |  |  | <0.001 |
| No | 11556 (98.7) | 1428901 (99.1) |  |
| Yes | 154 (1.3) | 12426 (0.9) |  |
| Diabetes |  |  | <0.001 |
| No | 10671 (91.1) | 1344689 (93.3) |  |
| Yes | 1039 (8.9) | 96638 (6.7) |  |

**Supplementary Table S3b: Characteristics of women with and without influenza giving birth in New South Wales Australia from 2001 to 2016**

|  | **Influenza** | | |
| --- | --- | --- | --- |
|  | **Yes**  **Number (%)** | **No**  **Number (%)** | **P value** |
| Age |  |  | 0.076 |
| 10-19 | 115 (4) | 48686 (3.4) |  |
| 20-24 | 390 (13.7) | 194533 (13.4) |  |
| 25-29 | 753 (26.4) | 395660 (27.3) |  |
| 30-35 | 920 (32.3) | 485755 (33.5) |  |
| 35-39 | 534 (18.7) | 265870 (18.3) |  |
| 40+ | 138 (4.8) | 59683 (4.1) |  |
| Smoking status |  |  | 1 |
| No | 2499 (87.7) | 1270142 (87.6) |  |
| Yes | 349 (12.2) | 177645 (12.2) |  |
| Unknown | 2 (0.1) | 2400 (0.2) |  |
| Remoteness of area of residence |  |  | 0.005 |
| Major city | 2330 (81.8) | 1155501 (79.7) |  |
| Inner regional | 389 (13.6) | 213828 (14.7) |  |
| Outer regional/Remote | 113 (4) | 63188 (4.4) |  |
| Unkown | 18 (0.6) | 17670 (1.2) |  |
| Socioeconomic status of area of residence |  |  | 0.483 |
| Q1 Most disadvantaged | 675 (23.7) | 351604 (24.2) |  |
| Q2 | 709 (24.9) | 351520 (24.2) |  |
| Q3 | 675 (23.7) | 351990 (24.3) |  |
| Q4 Least disadvantaged | 773 (27.1) | 377401 (26) |  |
| Unknown | 18 (0.6) | 17672 (1.2) |  |
| Previous pregnancy |  |  | <0.001 |
| No | 997 (35) | 618640 (42.7) |  |
| Yes | 1851 (64.9) | 830709 (57.3) |  |
| Unknown | 2 (0.1) | 838 (0.1) |  |
| Hospital of delivery |  |  | <0.001 |
| Public | 2372 (83.2) | 1127114 (77.7) |  |
| Private | 477 (16.7) | 323019 (22.3) |  |
| Unknown | 1 (0) | 54 (0) |  |
| Maternal country of birth |  |  | 0.009 |
| Australia | 2009 (70.5) | 989143 (68.2) |  |
| Overseas | 841 (29.5) | 461044 (31.8) |  |
| Weeks at first antenatal visit |  |  | 0.026 |
| 0-13 | 1991 (69.9) | 991643 (68.4) |  |
| 14-25 | 733 (25.7) | 374776 (25.8) |  |
| 26-45 | 102 (3.6) | 66729 (4.6) |  |
| Unknown | 24 (0.8) | 17039 (1.2) |  |
| Indigenous status |  |  | <0.001 |
| Non-indigenous | 2713 (95.2) | 1401322 (96.6) |  |
| Indigenous | 133 (4.7) | 46398 (3.2) |  |
| Unknown | 4 (0.1) | 2467 (0.2) |  |
| Hypertension |  |  | 0.211 |
| No | 2832 (99.4) | 1437625 (99.1) |  |
| Yes | 18 (0.6) | 12562 (0.9) |  |
| Diabetes |  |  | <0.001 |
| No | 2565 (90) | 1352795 (93.3) |  |
| Yes | 285 (10) | 97392 (6.7) |  |

**Supplementary Table S4: Association of acute respiratory infections during the pregnancy with various congenital anomalies**

| **Variables** | **Major congenital anomalies** | | **Cleft lip/palate** | | **Selected major cardiovascular anomalies** | | **All major cardiovascular anomalies (ICD AM Q20-25)** | |
| --- | --- | --- | --- | --- | --- | --- | --- | --- |
| **Acute respiratory infections** |  |  |  |  |  |  |  |  |
| No | 15486/1441327(1.1%) | Ref | 2222/1441327(0.2%) | Ref | 1724/1441327(0.1%) | Ref | 7671/1441327(0.5%) | Ref |
| 1^st^ trimester | 22/1547(1.4%) | 1.33 (0.88 - 2.02) | 1/1547(0.1%) | 0.43 (0.06 - 2.9) | 7/1547(0.5%) | 3.64 (1.73 - 7.66) | 14/1547(0.9%) | 1.68 (0.99 - 2.84) |
| 2^nd^/ 3^rd^ trimesters | 136/10163(1.3%) | 1.21 (1.02 - 1.44) | 19/10163(0.2%) | 1.19 (0.76 - 1.88) | 15/10163(0.1%) | 1.19 (0.72 - 1.99) | 85/10163(0.8%) | 1.47 (1.18 - 1.83) |
| **Age** |  |  |  |  |  |  |  |  |
| <20 | 617/48801(1.3%) | 1.07 (0.98 - 1.18) | 84/48801(0.2%) | 0.98 (0.77 - 1.24) | 76/48801(0.2%) | 1.26 (0.97 - 1.63) | 293/48801(0.6%) | 1.07 (0.93 - 1.22) |
| 20-24 | 2370/194923(1.2%) | 1.08 (1.03 - 1.14) | 309/194923(0.2%) | 0.96 (0.83 - 1.1) | 263/194923(0.1%) | 1.14 (0.97 - 1.33) | 1091/194923(0.6%) | 1.04 (0.96 - 1.12) |
| 25-29 | 4215/396413(1.1%) | Ref | 629/396413(0.2%) | Ref | 441/396413(0.1%) | Ref | 2006/396413(0.5%) | Ref |
| 30-35 | 4959/486675(1%) | 1 (0.96 - 1.05) | 715/486675(0.1%) | 0.95 (0.85 - 1.07) | 542/486675(0.1%) | 1.07 (0.94 - 1.22) | 2475/486675(0.5%) | 1.05 (0.99 - 1.11) |
| 35-39 | 2821/266404(1.1%) | 1.06 (1.01 - 1.11) | 411/266404(0.2%) | 0.99 (0.87 - 1.13) | 333/266404(0.1%) | 1.2 (1.03 - 1.4) | 1476/266404(0.6%) | 1.15 (1.07 - 1.24) |
| 40+ | 662/59821(1.1%) | 1.09 (1 - 1.19) | 94/59821(0.2%) | 1.01 (0.81 - 1.26) | 91/59821(0.2%) | 1.41 (1.12 - 1.79) | 429/59821(0.7%) | 1.44 (1.29 - 1.6) |
| **Smoking status** |  |  |  |  |  |  |  |  |
| No | 13517/1272641(1.1%) | Ref | 1900/1272641(0.1%) | Ref | 1528/1272641(0.1%) | Ref | 6655/1272641(0.5%) | Ref |
| Yes | 2103/177994(1.2%) | 1.01 (0.96 - 1.07) | 340/177994(0.2%) | 1.19 (1.05 - 1.36) | 216/177994(0.1%) | 0.79 (0.68 - 0.93) | 1099/177994(0.6%) | 1.05 (0.98 - 1.13) |
| Unknown | 24/2402(1%) |  | 2/2402(0.1%) |  | 2/2402(0.1%) |  | 16/2402(0.7%) |  |
| **Remoteness of area** |  |  |  |  |  |  |  |  |
| Major city | 12511/1157831(1.1%) | Ref | 1779/1157831(0.2%) | Ref | 1349/1157831(0.1%) | Ref | 6177/1157831(0.5%) | Ref |
| Inner regional | 2303/214217(1.1%) | 0.88 (0.84 - 0.92) | 347/214217(0.2%) | 0.93 (0.82 - 1.05) | 268/214217(0.1%) | 0.89 (0.77 - 1.03) | 1159/214217(0.5%) | 0.88 (0.82 - 0.94) |
| Outer regional/Remote | 665/63301(1.1%) | 0.84 (0.77 - 0.91) | 90/63301(0.1%) | 0.8 (0.64 - 1) | 91/63301(0.1%) | 0.99 (0.78 - 1.24) | 312/63301(0.5%) | 0.76 (0.68 - 0.86) |
| Unknown | 165/17688(0.9%) |  | 26/17688(0.1%) |  | 38/17688(0.2%) |  | 122/17688(0.7%) |  |
| **Socioeconomic status** |  |  |  |  |  |  |  |  |
| Q1 Most disadvantaged | 3992/352279(1.1%) | Ref | 532/352279(0.2%) | Ref | 446/352279(0.1%) | Ref | 1953/352279(0.6%) | Ref |
| Q2 | 3992/352229(1.1%) | 0.99 (0.94 - 1.03) | 609/352229(0.2%) | 1.09 (0.97 - 1.23) | 450/352229(0.1%) | 1.03 (0.9 - 1.17) | 1913/352229(0.5%) | 0.97 (0.91 - 1.04) |
| Q3 | 3802/352665(1.1%) | 0.95 (0.91 - 1) | 548/352665(0.2%) | 0.99 (0.87 - 1.12) | 396/352665(0.1%) | 0.95 (0.83 - 1.1) | 1921/352665(0.5%) | 0.99 (0.92 - 1.05) |
| Q4 Least disadvantaged | 3693/378174(1%) | 0.9 (0.85 - 0.94) | 527/378174(0.1%) | 0.9 (0.78 - 1.03) | 416/378174(0.1%) | 1.06 (0.91 - 1.23) | 1861/378174(0.5%) | 0.94 (0.87 - 1.01) |
| Unknown | 165/17690(0.9%) |  | 26/17690(0.1%) |  | 38/17690(0.2%) |  | 122/17690(0.7%) |  |
| **Previous pregnancy** |  |  |  |  |  |  |  |  |
| No | 6998/619637(1.1%) | Ref | 948/619637(0.2%) | Ref | 700/619637(0.1%) | Ref | 3210/619637(0.5%) | Ref |
| Yes | 8639/832560(1%) | 0.9 (0.87 - 0.94) | 1294/832560(0.2%) | 0.99 (0.9 - 1.08) | 1045/832560(0.1%) | 1.09 (0.98 - 1.21) | 4557/832560(0.5%) | 1.01 (0.96 - 1.06) |
| Unknown | 7/840(0.8%) |  |  |  | 1/840(0.1%) |  | 3/840(0.4%) |  |
| **Hospital of delivery** |  |  |  |  |  |  |  |  |
| Public | 12790/1129486(1.1%) | Ref | 1730/1129486(0.2%) | Ref | 1541/1129486(0.1%) | Ref | 6398/1129486(0.6%) | Ref |
| Private | 2854/323496(0.9%) | 0.77 (0.74 - 0.81) | 512/323496(0.2%) | 1.05 (0.94 - 1.18) | 205/323496(0.1%) | 0.43 (0.36 - 0.5) | 1372/323496(0.4%) | 0.73 (0.68 - 0.78) |
| Unknown |  |  |  |  |  |  |  |  |
| **Maternal country of birth** |  |  |  |  |  |  |  |  |
| Australia | 11048/991152(1.1%) | Ref | 1653/991152(0.2%) | Ref | 1239/991152(0.1%) | Ref | 5511/991152(0.6%) | Ref |
| Overseas | 4596/461885(1%) | 0.86 (0.83 - 0.89) | 589/461885(0.1%) | 0.79 (0.71 - 0.88) | 507/461885(0.1%) | 0.82 (0.73 - 0.92) | 2259/461885(0.5%) | 0.83 (0.79 - 0.88) |
| **First antenatal visit** |  |  |  |  |  |  |  |  |
| 0-13 | 10616/993634(1.1%) | Ref | 1513/993634(0.2%) | Ref | 1185/993634(0.1%) | Ref | 5257/993634(0.5%) | Ref |
| 14-25 | 3933/375509(1%) | 0.93 (0.9 - 0.97) | 575/375509(0.2%) | 1.02 (0.92 - 1.13) | 391/375509(0.1%) | 0.79 (0.7 - 0.89) | 1910/375509(0.5%) | 0.91 (0.87 - 0.97) |
| 26-45 | 856/66831(1.3%) | 1.14 (1.06 - 1.23) | 110/66831(0.2%) | 1.08 (0.88 - 1.31) | 145/66831(0.2%) | 1.63 (1.36 - 1.95) | 496/66831(0.7%) | 1.31 (1.19 - 1.44) |
| Unknown | 239/17063(1.4%) |  | 44/17063(0.3%) |  | 25/17063(0.1%) |  | 107/17063(0.6%) |  |
| **Indigenous status** |  |  |  |  |  |  |  |  |
| Non-indigenous | 15050/1404035(1.1%) | Ref | 2148/1404035(0.2%) | Ref | 1662/1404035(0.1%) | Ref | 7406/1404035(0.5%) | Ref |
| Indigenous | 568/46531(1.2%) | 1.03 (0.94 - 1.13) | 91/46531(0.2%) | 1.19 (0.95 - 1.49) | 83/46531(0.2%) | 1.23 (0.96 - 1.57) | 350/46531(0.8%) | 1.28 (1.14 - 1.44) |
| Unknown | 26/2471(1.1%) |  | 3/2471(0.1%) |  | 1/2471(0%) |  | 14/2471(0.6%) |  |
| **Hypertension** |  |  |  |  |  |  |  |  |
| No | 15444/1440457(1.1%) | Ref | 2219/1440457(0.2%) | Ref | 1729/1440457(0.1%) | Ref | 7665/1440457(0.5%) | Ref |
| Yes | 200/12580(1.6%) | 1.43 (1.24 - 1.65) | 23/12580(0.2%) | 1.19 (0.79 - 1.79) | 17/12580(0.1%) | 1.05 (0.65 - 1.69) | 105/12580(0.8%) | 1.46 (1.2 - 1.78) |
| **Diabetes*** |  |  |  |  |  |  |  |  |
| No | 14422/1355360(1.1%) | Ref | 2086/1355360(0.2%) | Ref | 1581/1355360(0.1%) | Ref | 7081/1355360(0.5%) | Ref |
| Yes | 1222/97677(1.3%) | 1.17 (1.1 - 1.24) | 156/97677(0.2%) | 1.06 (0.9 - 1.25) | 165/97677(0.2%) | 1.4 (1.19 - 1.66) | 689/97677(0.7%) | 1.31 (1.21 - 1.42) |

***** Pregestational /gestational diabetes

**Supplementary Table S5: Association influenza during the pregnancy with various congenital anomalies**

| **Variables** | **Major congenital anomalies** | | **Cleft lip/palate** | | **Selected major cardiovascular anomalies** | | **All major cardiovascular anomalies (ICD AM Q20-25)** | |
| --- | --- | --- | --- | --- | --- | --- | --- | --- |
| **Influenza** |  |  |  |  |  |  |  |  |
| No | 15612/1450187(1.1%) | Ref | 2239/1450187(0.2%) | Ref | 1739/1450187(0.1%) | Ref | 7745/1450187(0.5%) | Ref |
| 1^st^ trimester | 4/399(1%) | 0.95 (0.36 - 2.54) | 1/399(0.3%) | 1.68 (0.23 - 12.18) | 1/399(0.3%) | 2.04 (0.29 - 14.42) | 2/399(0.5%) | 0.95 (0.24 - 3.78) |
| 2^nd^/ 3^rd^ trimesters | 28/2451(1.1%) | 1.02 (0.7 - 1.5) | 2/2451(0.1%) | 0.53 (0.13 - 2.15) | 6/2451(0.2%) | 1.97 (0.89 - 4.39) | 23/2451(0.9%) | 1.66 (1.09 - 2.52) |
| **Age** |  |  |  |  |  |  |  |  |
| <20 | 617/48801(1.3%) | 1.08 (0.98 - 1.18) | 84/48801(0.2%) | 0.98 (0.77 - 1.24) | 76/48801(0.2%) | 1.26 (0.97 - 1.64) | 293/48801(0.6%) | 1.07 (0.94 - 1.22) |
| 20-24 | 2370/194923(1.2%) | 1.08 (1.03 - 1.14) | 309/194923(0.2%) | 0.96 (0.83 - 1.1) | 263/194923(0.1%) | 1.14 (0.97 - 1.33) | 1091/194923(0.6%) | 1.04 (0.96 - 1.12) |
| 25-29 | 4215/396413(1.1%) | Ref | 629/396413(0.2%) | Ref | 441/396413(0.1%) | Ref | 2006/396413(0.5%) | Ref |
| 30-35 | 4959/486675(1%) | 1 (0.96 - 1.05) | 715/486675(0.1%) | 0.95 (0.85 - 1.07) | 542/486675(0.1%) | 1.07 (0.94 - 1.22) | 2475/486675(0.5%) | 1.05 (0.99 - 1.11) |
| 35-39 | 2821/266404(1.1%) | 1.06 (1.01 - 1.11) | 411/266404(0.2%) | 0.99 (0.87 - 1.13) | 333/266404(0.1%) | 1.2 (1.03 - 1.4) | 1476/266404(0.6%) | 1.15 (1.07 - 1.24) |
| 40+ | 662/59821(1.1%) | 1.09 (1 - 1.19) | 94/59821(0.2%) | 1.01 (0.81 - 1.26) | 91/59821(0.2%) | 1.41 (1.12 - 1.79) | 429/59821(0.7%) | 1.44 (1.29 - 1.6) |
| **Smoking status** |  |  |  |  |  |  |  |  |
| No | 13517/1272641(1.1%) | Ref | 1900/1272641(0.1%) | Ref | 1528/1272641(0.1%) | Ref | 6655/1272641(0.5%) | Ref |
| Yes | 2103/177994(1.2%) | 1.02 (0.96 - 1.07) | 340/177994(0.2%) | 1.19 (1.05 - 1.36) | 216/177994(0.1%) | 0.79 (0.68 - 0.93) | 1099/177994(0.6%) | 1.05 (0.98 - 1.13) |
| Unknown | 24/2402(1%) | Ref | 2/2402(0.1%) | Ref | 2/2402(0.1%) | Ref | 16/2402(0.7%) | Ref |
| **Remoteness of area** |  |  |  |  |  |  |  |  |
| Major city | 12511/1157831(1.1%) | Ref | 1779/1157831(0.2%) | Ref | 1349/1157831(0.1%) | Ref | 6177/1157831(0.5%) | Ref |
| Inner regional | 2303/214217(1.1%) | 0.88 (0.84 - 0.92) | 347/214217(0.2%) | 0.93 (0.82 - 1.05) | 268/214217(0.1%) | 0.89 (0.77 - 1.03) | 1159/214217(0.5%) | 0.88 (0.82 - 0.94) |
| Outer regional/Remote | 665/63301(1.1%) | 0.84 (0.77 - 0.91) | 90/63301(0.1%) | 0.8 (0.64 - 1) | 91/63301(0.1%) | 0.99 (0.79 - 1.25) | 312/63301(0.5%) | 0.77 (0.68 - 0.86) |
| Unknown | 165/17688(0.9%) |  | 26/17688(0.1%) |  | 38/17688(0.2%) |  | 122/17688(0.7%) |  |
| **Socioeconomic status** |  |  |  |  |  |  |  |  |
| Q1 Most disadvantaged | 3992/352279(1.1%) | Ref | 532/352279(0.2%) | Ref | 446/352279(0.1%) | Ref | 1953/352279(0.6%) | Ref |
| Q2 | 3992/352229(1.1%) | 0.99 (0.94 - 1.03) | 609/352229(0.2%) | 1.09 (0.97 - 1.23) | 450/352229(0.1%) | 1.03 (0.9 - 1.17) | 1913/352229(0.5%) | 0.97 (0.91 - 1.04) |
| Q3 | 3802/352665(1.1%) | 0.95 (0.91 - 1) | 548/352665(0.2%) | 0.99 (0.87 - 1.12) | 396/352665(0.1%) | 0.95 (0.83 - 1.1) | 1921/352665(0.5%) | 0.99 (0.92 - 1.05) |
| Q4 Least disadvantaged | 3693/378174(1%) | 0.9 (0.85 - 0.94) | 527/378174(0.1%) | 0.9 (0.78 - 1.03) | 416/378174(0.1%) | 1.06 (0.91 - 1.23) | 1861/378174(0.5%) | 0.94 (0.87 - 1.01) |
| Unknown | 165/17690(0.9%) |  | 26/17690(0.1%) |  | 38/17690(0.2%) |  | 122/17690(0.7%) |  |
| **Previous pregnancy** |  |  |  |  |  |  |  |  |
| No | 6998/619637(1.1%) | Ref | 948/619637(0.2%) | Ref | 700/619637(0.1%) | Ref | 3210/619637(0.5%) | Ref |
| Yes | 8639/832560(1%) | 0.91 (0.87 - 0.94) | 1294/832560(0.2%) | 0.99 (0.9 - 1.08) | 1045/832560(0.1%) | 1.09 (0.98 - 1.21) | 4557/832560(0.5%) | 1.01 (0.97 - 1.06) |
| Unknown | 7/840(0.8%) |  |  |  | 1/840(0.1%) |  | 3/840(0.4%) |  |
| **Hospital of delivery** |  |  |  |  |  |  |  |  |
| Public | 12790/1129486(1.1%) | Ref | 1730/1129486(0.2%) | Ref | 1541/1129486(0.1%) | Ref | 6398/1129486(0.6%) | Ref |
| Private | 2854/323496(0.9%) | 0.77 (0.74 - 0.81) | 512/323496(0.2%) | 1.05 (0.94 - 1.18) | 205/323496(0.1%) | 0.43 (0.36 - 0.5) | 1372/323496(0.4%) | 0.73 (0.68 - 0.78) |
| Unknown |  |  |  |  |  |  |  |  |
| **Maternal country of birth** |  |  |  |  |  |  |  |  |
| Australia | 11048/991152(1.1%) | Ref | 1653/991152(0.2%) | Ref | 1239/991152(0.1%) | Ref | 5511/991152(0.6%) | Ref |
| Overseas | 4596/461885(1%) | 0.86 (0.83 - 0.89) | 589/461885(0.1%) | 0.79 (0.71 - 0.88) | 507/461885(0.1%) | 0.82 (0.73 - 0.92) | 2259/461885(0.5%) | 0.83 (0.79 - 0.88) |
| **First antenatal visit** |  |  |  |  |  |  |  |  |
| 0-13 | 10616/993634(1.1%) | Ref | 1513/993634(0.2%) | Ref | 1185/993634(0.1%) | Ref | 5257/993634(0.5%) | Ref |
| 14-25 | 3933/375509(1%) | 0.93 (0.9 - 0.97) | 575/375509(0.2%) | 1.02 (0.92 - 1.13) | 391/375509(0.1%) | 0.79 (0.7 - 0.89) | 1910/375509(0.5%) | 0.91 (0.86 - 0.97) |
| 26-45 | 856/66831(1.3%) | 1.14 (1.06 - 1.23) | 110/66831(0.2%) | 1.08 (0.88 - 1.31) | 145/66831(0.2%) | 1.63 (1.36 - 1.95) | 496/66831(0.7%) | 1.31 (1.19 - 1.44) |
| Unknown | 239/17063(1.4%) |  | 44/17063(0.3%) |  | 25/17063(0.1%) |  | 107/17063(0.6%) |  |
| **Indigenous status** |  |  |  |  |  |  |  |  |
| Non-indigenous | 15050/1404035(1.1%) | Ref | 2148/1404035(0.2%) | Ref | 1662/1404035(0.1%) | Ref | 7406/1404035(0.5%) | Ref |
| Indigenous | 568/46531(1.2%) | 1.04 (0.94 - 1.13) | 91/46531(0.2%) | 1.19 (0.95 - 1.49) | 83/46531(0.2%) | 1.23 (0.96 - 1.58) | 350/46531(0.8%) | 1.28 (1.14 - 1.45) |
| Unknown | 26/2471(1.1%) |  | 3/2471(0.1%) |  | 1/2471(0%) |  | 14/2471(0.6%) |  |
| **Hypertension** |  |  |  |  |  |  |  |  |
| No | 15444/1440457(1.1%) | Ref | 2219/1440457(0.2%) | Ref | 1729/1440457(0.1%) | Ref | 7665/1440457(0.5%) | Ref |
| Yes | 200/12580(1.6%) | 1.43 (1.24 - 1.65) | 23/12580(0.2%) | 1.19 (0.79 - 1.79) | 17/12580(0.1%) | 1.05 (0.65 - 1.69) | 105/12580(0.8%) | 1.47 (1.21 - 1.78) |
| **Diabetes*** |  |  |  |  |  |  |  |  |
| No | 14422/1355360(1.1%) | Ref | 2086/1355360(0.2%) | Ref | 1581/1355360(0.1%) | Ref | 7081/1355360(0.5%) | Ref |
| Yes | 1222/97677(1.3%) | 1.17 (1.1 - 1.24) | 156/97677(0.2%) | 1.06 (0.9 - 1.25) | 165/97677(0.2%) | 1.4 (1.19 - 1.66) | 689/97677(0.7%) | 1.31 (1.21 - 1.42) |

***** Pregestational /gestational diabetes

**Supplementary Table S6: Association of high risk infections* during the pregnancy with various congenital anomalies**

| **Variables** | **Major congenital anomalies** | | **Cleft lip/palate** | | **Selected major cardiovascular anomalies** | | **All major cardiovascular anomalies (ICD AM Q20-25)** | |
| --- | --- | --- | --- | --- | --- | --- | --- | --- |
| **High risk infections** |  |  |  |  |  |  |  |  |
| No | 15630/1452025(1.1%) | Ref | 2240/1452025(0.2%) | Ref | 1745/1452025(0.1%) | Ref | 7758/1452025(0.5%) | Ref |
| 1^st^ trimester | 1/129(0.8%) | 0.72 (0.1 - 5.27) | 0/129(0%) |  | 0/129(0%) |  | 1/129(0.8%) | 1.41 (0.19 - 10.26) |
| 2^nd^/ 3^rd^ trimesters | 13/883(1.5%) | 1.27 (0.72 - 2.23) | 2/883(0.2%) | 1.5 (0.38 - 5.95) | 1/883(0.1%) | 0.9 (0.13 - 6.35) | 11/883(1.2%) | 2.07 (1.11 - 3.86) |
| **Age** |  |  |  |  |  |  |  |  |
| <20 | 617/48801(1.3%) | 1.08 (0.98 - 1.18) | 84/48801(0.2%) | 0.98 (0.77 - 1.24) | 76/48801(0.2%) | 1.26 (0.97 - 1.64) | 293/48801(0.6%) | 1.07 (0.94 - 1.22) |
| 20-24 | 2370/194923(1.2%) | 1.08 (1.03 - 1.14) | 309/194923(0.2%) | 0.96 (0.83 - 1.1) | 263/194923(0.1%) | 1.14 (0.97 - 1.33) | 1091/194923(0.6%) | 1.04 (0.96 - 1.12) |
| 25-29 | 4215/396413(1.1%) | Ref | 629/396413(0.2%) | Ref | 441/396413(0.1%) | Ref | 2006/396413(0.5%) | Ref |
| 30-35 | 4959/486675(1%) | 1 (0.96 - 1.05) | 715/486675(0.1%) | 0.95 (0.85 - 1.07) | 542/486675(0.1%) | 1.07 (0.94 - 1.22) | 2475/486675(0.5%) | 1.05 (0.99 - 1.11) |
| 35-39 | 2821/266404(1.1%) | 1.06 (1.01 - 1.11) | 411/266404(0.2%) | 0.99 (0.87 - 1.13) | 333/266404(0.1%) | 1.2 (1.03 - 1.4) | 1476/266404(0.6%) | 1.15 (1.07 - 1.24) |
| 40+ | 662/59821(1.1%) | 1.09 (1 - 1.19) | 94/59821(0.2%) | 1.01 (0.81 - 1.26) | 91/59821(0.2%) | 1.42 (1.12 - 1.79) | 429/59821(0.7%) | 1.44 (1.29 - 1.6) |
| **Smoking status** |  |  |  |  |  |  |  |  |
| No | 13517/1272641(1.1%) | Ref | 1900/1272641(0.1%) | Ref | 1528/1272641(0.1%) | Ref | 6655/1272641(0.5%) | Ref |
| Yes | 2103/177994(1.2%) | 1.02 (0.96 - 1.07) | 340/177994(0.2%) | 1.19 (1.05 - 1.36) | 216/177994(0.1%) | 0.79 (0.68 - 0.93) | 1099/177994(0.6%) | 1.05 (0.98 - 1.13) |
| Unknown | 24/2402(1%) |  | 2/2402(0.1%) |  | 2/2402(0.1%) |  | 16/2402(0.7%) |  |
| **Remoteness of area** |  |  |  |  |  |  |  |  |
| Major city | 12511/1157831(1.1%) | Ref | 1779/1157831(0.2%) | Ref | 1349/1157831(0.1%) | Ref | 6177/1157831(0.5%) | Ref |
| Inner regional | 2303/214217(1.1%) | 0.88 (0.84 - 0.92) | 347/214217(0.2%) | 0.93 (0.82 - 1.05) | 268/214217(0.1%) | 0.89 (0.77 - 1.03) | 1159/214217(0.5%) | 0.88 (0.82 - 0.94) |
| Outer regional/Remote | 665/63301(1.1%) | 0.84 (0.77 - 0.91) | 90/63301(0.1%) | 0.8 (0.64 - 1) | 91/63301(0.1%) | 0.99 (0.79 - 1.25) | 312/63301(0.5%) | 0.77 (0.68 - 0.86) |
| Unknown | 165/17688(0.9%) |  | 26/17688(0.1%) |  | 38/17688(0.2%) |  | 122/17688(0.7%) |  |
| **Socioeconomic status** |  |  |  |  |  |  |  |  |
| Q1 Most disadvantaged | 3992/352279(1.1%) | Ref | 532/352279(0.2%) | Ref | 446/352279(0.1%) | Ref | 1953/352279(0.6%) | Ref |
| Q2 | 3992/352229(1.1%) | 0.99 (0.94 - 1.03) | 609/352229(0.2%) | 1.09 (0.97 - 1.23) | 450/352229(0.1%) | 1.03 (0.9 - 1.17) | 1913/352229(0.5%) | 0.97 (0.91 - 1.04) |
| Q3 | 3802/352665(1.1%) | 0.95 (0.91 - 1) | 548/352665(0.2%) | 0.99 (0.87 - 1.12) | 396/352665(0.1%) | 0.95 (0.83 - 1.1) | 1921/352665(0.5%) | 0.99 (0.92 - 1.05) |
| Q4 Least disadvantaged | 3693/378174(1%) | 0.9 (0.85 - 0.94) | 527/378174(0.1%) | 0.9 (0.78 - 1.03) | 416/378174(0.1%) | 1.06 (0.91 - 1.23) | 1861/378174(0.5%) | 0.94 (0.87 - 1.01) |
| Unknown | 165/17690(0.9%) |  | 26/17690(0.1%) |  | 38/17690(0.2%) |  | 122/17690(0.7%) |  |
| **Previous pregnancy** |  |  |  |  |  |  |  |  |
| No | 6998/619637(1.1%) | Ref | 948/619637(0.2%) | Ref | 700/619637(0.1%) | Ref | 3210/619637(0.5%) | Ref |
| Yes | 8639/832560(1%) | 0.91 (0.87 - 0.94) | 1294/832560(0.2%) | 0.99 (0.9 - 1.08) | 1045/832560(0.1%) | 1.09 (0.98 - 1.21) | 4557/832560(0.5%) | 1.01 (0.97 - 1.06) |
| Unknown | 7/840(0.8%) |  |  |  | 1/840(0.1%) |  | 3/840(0.4%) |  |
| **Hospital of delivery** |  |  |  |  |  |  |  |  |
| Public | 12790/1129486(1.1%) | Ref | 1730/1129486(0.2%) | Ref | 1541/1129486(0.1%) | Ref | 6398/1129486(0.6%) | Ref |
| Private | 2854/323496(0.9%) | 0.77 (0.74 - 0.81) | 512/323496(0.2%) | 1.05 (0.94 - 1.18) | 205/323496(0.1%) | 0.43 (0.36 - 0.5) | 1372/323496(0.4%) | 0.73 (0.68 - 0.78) |
| Unknown |  |  |  |  |  |  |  |  |
| **Maternal country of birth** |  |  |  |  |  |  |  |  |
| Australia | 11048/991152(1.1%) | Ref | 1653/991152(0.2%) | Ref | 1239/991152(0.1%) | Ref | 5511/991152(0.6%) | Ref |
| Overseas | 4596/461885(1%) | 0.86 (0.83 - 0.89) | 589/461885(0.1%) | 0.79 (0.71 - 0.88) | 507/461885(0.1%) | 0.82 (0.73 - 0.92) | 2259/461885(0.5%) | 0.83 (0.79 - 0.88) |
| **First antenatal visit** |  |  |  |  |  |  |  |  |
| 0-13 | 10616/993634(1.1%) | Ref | 1513/993634(0.2%) | Ref | 1185/993634(0.1%) | Ref | 5257/993634(0.5%) | Ref |
| 14-25 | 3933/375509(1%) | 0.93 (0.9 - 0.97) | 575/375509(0.2%) | 1.02 (0.92 - 1.13) | 391/375509(0.1%) | 0.79 (0.7 - 0.89) | 1910/375509(0.5%) | 0.91 (0.86 - 0.96) |
| 26-45 | 856/66831(1.3%) | 1.14 (1.06 - 1.23) | 110/66831(0.2%) | 1.08 (0.88 - 1.31) | 145/66831(0.2%) | 1.63 (1.36 - 1.95) | 496/66831(0.7%) | 1.31 (1.19 - 1.44) |
| Unknown | 239/17063(1.4%) |  | 44/17063(0.3%) |  | 25/17063(0.1%) |  | 107/17063(0.6%) |  |
| **Indigenous status** |  |  |  |  |  |  |  |  |
| Non-indigenous | 15050/1404035(1.1%) | Ref | 2148/1404035(0.2%) | Ref | 1662/1404035(0.1%) | Ref | 7406/1404035(0.5%) | Ref |
| Indigenous | 568/46531(1.2%) | 1.04 (0.94 - 1.13) | 91/46531(0.2%) | 1.19 (0.95 - 1.49) | 83/46531(0.2%) | 1.23 (0.96 - 1.58) | 350/46531(0.8%) | 1.28 (1.14 - 1.45) |
| Unknown | 26/2471(1.1%) |  | 3/2471(0.1%) |  | 1/2471(0%) |  | 14/2471(0.6%) |  |
| **Hypertension** |  |  |  |  |  |  |  |  |
| No | 15444/1440457(1.1%) | Ref | 2219/1440457(0.2%) | Ref | 1729/1440457(0.1%) | Ref | 7665/1440457(0.5%) | Ref |
| Yes | 200/12580(1.6%) | 1.43 (1.24 - 1.65) | 23/12580(0.2%) | 1.19 (0.79 - 1.79) | 17/12580(0.1%) | 1.05 (0.65 - 1.69) | 105/12580(0.8%) | 1.46 (1.2 - 1.78) |
| **Diabetes**** |  |  |  |  |  |  |  |  |
| No | 14422/1355360(1.1%) | Ref | 2086/1355360(0.2%) | Ref | 1581/1355360(0.1%) | Ref | 7081/1355360(0.5%) | Ref |
| Yes | 1222/97677(1.3%) | 1.17 (1.1 - 1.24) | 156/97677(0.2%) | 1.06 (0.9 - 1.25) | 165/97677(0.2%) | 1.41 (1.19 - 1.66) | 689/97677(0.7%) | 1.31 (1.21 - 1.42) |

***** Included cytomegalovirus, rubella, herpes simplex, herpes zoster, toxoplasmosis, syphilis, chickenpox (varicella), zika.

****** Pregestational /gestational diabetes
